# The cryopreservation process induces alterations in proteins associated with bull sperm quality: The equilibration process could be a probable critical control point

**DOI:** 10.3389/fendo.2022.1064956

**Published:** 2022-12-09

**Authors:** Ramasamy Arunkumar, Arumugam Kumaresan, Manish Kumar Sinha, Kamaraj Elango, John Peter Ebenezer Samuel King, Pradeep Nag, Thirumalaisamy Karuthadurai, Rubina Kumari Baithalu, Tushar Kumar Mohanty, Rakesh Kumar, Tirtha Kumar Datta

**Affiliations:** ^1^ Theriogenology Laboratory, Southern Regional Station of ICAR-National Dairy Research Institute, Bengaluru, India; ^2^ Animal Reproduction, Gynaecology and Obstetrics, ICAR-National Dairy Research Institute, Karnal, India; ^3^ Animal Genomics Laboratory, Indian Council for Agricultural Research (ICAR)-National Dairy Research Institute, Karnal, India

**Keywords:** proteome, stages of cryopreservation, LC-MS/MS, bioinformatics, bull spermatozoa

## Abstract

The present study quantitatively characterized the proteomic changes in bull spermatozoa induced by the cryopreservation process. We performed high-throughput comparative global proteomic profiling of freshly ejaculated (before cryopreservation), equilibrated (refrigerated storage; during cryopreservation), and frozen (ultralow temperature; after cryopreservation) bull spermatozoa. Using the liquid chromatography–mass spectrometry (LC-MS/MS) technique, a total of 1,692, 1,415, and 1,286 proteins were identified in fresh, equilibrated, and cryopreserved spermatozoa, respectively. When the proteome of fresh spermatozoa was compared with equilibrated spermatozoa, we found that 166 proteins were differentially expressed. When equilibrated spermatozoa were compared with cryopreserved spermatozoa, we found that 147 proteins were differentially expressed between them. Similarly, we found that 156 proteins were differentially expressed between fresh and cryopreserved spermatozoa. Among these proteins, the abundance of 105 proteins was lowered during the equilibration process itself, while the abundance of 43 proteins was lowered during ultralow temperature preservation. Remarkably, the equilibration process lowered the abundance of sperm proteins involved in energy metabolism, structural integrity, and DNA repair and increased the abundance of proteins associated with proteolysis and protein degradation. The abundance of sperm proteins associated with metabolism, cGMP-PKG (cyclic guanosine 3′,5′-monophosphate-dependent protein kinase G) signaling, and regulation of the actin cytoskeleton was also altered during the equilibration process. Collectively, the present study showed that the equilibration step in the bull sperm cryopreservation process was the critical point for sperm proteome, during which a majority of proteomic alterations in sperm occurred. These findings are valuable for developing efficient protocols to minimize protein damage and to improve the quality and fertility of cryopreserved bull spermatozoa.

## Introduction

Artificial insemination using cryopreserved semen has undeniably contributed to the genetic improvement and productivity enhancement of cattle (1). The long-term *ex-situ* conservation of superior germplasm by cryopreservation enables the improvement of genetic gain even after the sire’s death (2). By using cryopreserved spermatozoa, the first cattle calf was produced in 1951 (3); since then, the technique was rapidly established worldwide. In spite of several developments in semen cryopreservation, a large number of spermatozoa are rendered immotile during this process; the lifespan and fertilizing potential of the motile sperm population in cryopreserved semen are also altered (4, 5). It is reasonably understood that the cryotolerance of the spermatozoa varied with individual bulls. The spermatozoa from different bulls respond differently to the changing environment during the process of cryopreservation (6, 7). Furthermore, increasing lines of evidence indicate that functional attributes like membrane integrity (8), acrosomal integrity (9), capacitation (10), and oviduct/zona binding ability (11) are altered in the cryopreserved spermatozoa. Although it is well proven that sperm functions are altered by the process of cryopreservation, the exact reason(s) for decreased fertility with cryopreserved spermatozoa still remains obscure.

The transcription and translation processes in mature spermatozoa are still debatable. Few studies evaluated the molecular alterations in cryopreserved spermatozoa and reported alterations in mRNA profiles, chromatin remodeling, DNA methylation, and post-translational histone modifications (12–14). Although genomics and transcriptomics have achieved new heights and are of extensive importance, they do not reveal temporal and spatial protein expression or protein levels and post-translational modifications (15, 16). Therefore, studies are required to identify the effect of the cryopreservation process on sperm proteins, which are indispensable for sperm binding to the oviduct or oocyte and for fertilization. Among the several molecular alterations that take place during the process of cryopreservation, sperm protein architectural alterations are extremely important. It is well proven that sperm proteins are important for timely capacitation (17), hyperactivated motility (18), acrosome reaction (19), sperm–oviduct binding, sperm–oocyte binding (20), and fertilization (21, 22). Protein alterations in the sperm could be the major cause of cryopreservation-induced premature capacitation and acrosome exocytosis, which decreases the fertilizing capacity of cryopreserved spermatozoa (23). Few studies have indicated that the cryopreservation process induces differential remodeling of the proteome in mammalian spermatozoa (24). Therefore, analysis of sperm proteomic alterations during various stages of cryopreservation is a promising way to understand the reasons behind the reduced fertility with cryopreserved spermatozoa. Furthermore, it would help us to tailor the cryopreservation protocol for bull spermatozoa with the aim to improve the quality and fertility of cryopreserved spermatozoa.

The process of cryopreservation involves several steps; the most prominent are the process of equilibration and ultralow freezing. In order to identify the critical step of cryopreservation, which induces differential remodeling of the sperm proteome, the sperm proteome needs to be analyzed at different stages of cryopreservation. Once the critical point is identified, quality control measures can be taken up for the development of an efficient cryopreservation technology that protects bull sperm proteome. With this background in mind, the aim of the current study was to find out the potential alterations of bull sperm proteome at different stages of the cryopreservation process. Using the high-throughput proteomic profiling technique, we report here the proteomic profile of fresh, equilibrated, and cryopreserved bull spermatozoa and the proteomic alterations in spermatozoa at each stage of the cryopreservation process.

## Materials and methods

### Experimental animals

The present investigation was conducted on Holstein Friesian crossbred bulls maintained at the Artificial Breeding Research Centre, ICAR-National Dairy Research Institute, Karnal, India. Six ejaculates from three crossbred bulls were utilized for the study. All the experimental bulls have qualified the breeding soundness evaluation and were routinely used for artificial breeding. The age of the bulls ranged between 4 and 6 years, and they were housed under a loose housing system in individual pens (30′ × 10′) on concrete floor. The bulls were fed with 2.5 kg of concentrate ration containing 21% crude protein and 70% total digestible nutrients. Seasonal green fodders, including maize, cowpea, berseem, and jowar, as well as a blend of maize and oat silage, were made *ad libitum* to the animals. The bulls were exercised in the rotatory exerciser on the day before semen collection (twice a week), in order to preserve the sexual activity and to ensure consistent quality of semen. All the experiments were conducted in accordance with the guidelines and regulations laid down and duly approved by the Institute Animal Ethics Committee (CPCSEA/IAEC/LA/SRS-ICAR-NDRI-2019/No.04).

### Semen collection and sample preparation

Ejaculates were collected from the bulls using the Danish model standard artificial vagina (AV, 14") (IMV Technologies, France). The temperature of the artificial vagina (AV) was maintained between 42°C and 45°C with sufficient pressure and proper lubrication with sterilized K-Y lubricating jelly (Johnson & Johnson Co., USA). After preliminary evaluation, two ejaculates from each bull were selected based on mass activity (+3.0 and above in the 0–5 scale), progressive motility (≥80%), and sperm concentration (>800 million/ml) and pooled before further processing. The present study was designed to analyze the proteomic profiles of fresh, equilibrated, and cryopreserved sperm samples prepared from the same animal ejaculates. In order to do that, the pooled ejaculates from each animal were divided into two aliquots. One aliquot was used as fresh, while another aliquot was processed into equilibration followed by ultralow freezing. For fresh sample preparation, seminal plasma was separated from spermatozoa by centrifugation (1,000×*g* for 15 min at 4°C), and the sperm pellet obtained was washed twice with 1 ml of PBS (pH 7.4, 10× PBS containing 1.37 M of NaCl, 27 mM of KCl, 100 mM of Na_2_HPO_4_, and 18 mM of KH_2_PO_4_) (Sigma Aldrich, USA) at 900×*g* for 5 min at 4°C. The supernatant was discarded and the pellet was purified by double layer 90%–45% discontinuous Percoll gradient centrifugation (25) to eliminate contaminating substances like epithelium cells. Protease inhibitor cocktail [1% (v/v); Amresco, USA, Cat. No. M221] was added to spermatozoa and snap-frozen in liquid nitrogen until further use (26). For the preparation of equilibrated and cryopreserved sperm samples, another aliquot of semen was extended with an egg yolk-free commercial extender (AndroMed*^®^*, Minitube Animal Reproduction Technologies, Germany, Cat. No. 13503/0201). The extended semen was filled in French mini straws (0.25 ml) with the help of an automatic straw filling and sealing machine (MRS 3, IMV France) and equilibrated in a single-layer horizontal position on the straw rack for 4 h at 5°C. After equilibration, the straws were cut and the semen sample was centrifuged at 1,000×*g* for 15 min at 4°C to remove the extender. After washing, the Percoll-selected spermatozoa were snap-frozen in liquid nitrogen after the addition of 1% (v/v) protease cocktail inhibitor. For the preparation of the cryopreserved sample, a portion of equilibrated semen straws was exposed to liquid nitrogen vapor by keeping the straws 4 cm above the liquid nitrogen level in the wide opened LN2 container for 5 min. The straws were then placed into precooled goblets using precooled forceps before being submerged in liquid nitrogen. After 24 h of cryopreservation, semen straws were thawed at 37°C for 30 s. Cryopreservation was considered successful when at least 50% of the spermatozoa remained motile after thawing. The frozen-thawed semen was washed and the Percoll-selected spermatozoa were snap-frozen in liquid nitrogen after the addition of 1% (v/v) protease cocktail inhibitor. The mean (± SE) sperm motility (%) in fresh, equilibrated, and cryopreserved semen was 83.5 ± 1.5, 71.6 ± 1.4 and 52.5 ± 1.6 percentage, respectively.

### Protein extraction

For the isolation of protein, 50 million spermatozoa were taken, into which 100 µl of 50 mM NH_4_HCO_3_ was added and incubated at room temperature for 30 min. Then, the samples were sonicated for 10 min and centrifuged at 200×*g* for 10 min at 4°C. To these samples, sodium dodecyl sulfate (SDS) was added, and in each step, the samples were incubated at room temperature for 10 min, followed by sonication and centrifugation. The supernatant was taken further for SDS-PAGE for quality checking.

### Gel electrophoresis and protein digestion

The glass plates (with 1.5 mm spacer) were cleaned thoroughly and then fixed into the casting assembly. The resolving gel (appendix) was prepared and poured in between the glass plates up to the level, 2 cm below the top, and then iso-butanol was slowly added so as to just cover the top surface of the resolving gel to avoid oxygen and to make the upper surface linear. The resolving gel was allowed to polymerize for 15 min. The iso-butanol was removed and washed twice with triple distilled water. Finally, the stacking gel (5%) solution was poured up to the top; the comb was fixed and kept for 15–20 min. The sperm proteins were separated by the SDS-PAGE method. The sample preparation involved the denaturation of proteins, by boiling the sample with 2× lysis buffer in a 1:1 ratio for 5 min in the presence of 2-mercaptoethanol and SDS. After boiling, the samples were loaded into the wells along with molecular weight markers as the reference standard. Electrophoresis was carried out at a constant current of 100 mA in the stacking gel and at 100 mA in the resolving gel. The electrophoresis was stopped when the dye front reached about 10 mm above the bottom, and the glass plates were disassembled. The stacking gel portion was cut and the resolving gel was transferred to a fixative for 4 h, and furthermore, the gels were silver-stained to observe the bands. Finally, the gel was scanned with an Epson Expression 11000XL Scanner (**Supplementary Figure 1**).

For the in-solution digestion, 100 μg of the sample was taken and diluted with 50 mM of NH_4_HCO_3_. This sample was treated with 100 mM of DTT at 95°C for 1 h followed by 250 mM of IDA at room temperature in the dark for 45 min and then digested with trypsin and incubated overnight at 37°C. The resulting sample was vacuum-dried and dissolved in 50 µl of 0.1% formic acid in water. This solution was centrifuged at 10,000×*g*, and the supernatant was collected into a separate tube. For label-free quantitation, 3 runs per sample were carried out. The samples were cleaned up using ZipTip as per the manufacturer’s protocol.

### Mass spectrometry analysis of peptide mixtures

Samples (10 µl) were injected into the C18 UPLC column (Waters UPLC system). The separation of all the samples was performed on ACQUITY UPLC BEH C18 column (Waters, UK) (75 µm × 150 mm × 1.7 µm). A gradient elution program was run for the chromatographic separation with mobile phase A (0.1% formic acid in water) and mobile phase B (0.1% formic acid in acetonitrile) followed by analysis on the Q-TOF instrument for MS and MS/MS [Synapt G2 Mass Spectrometer equipped with an electrospray ionization (ESI) source]. Sample analysis was performed in the positive mode. The experimental instrument parameters include polarity- ES+, analyser- resolution mode, capillary (kV)- 3.5000, source temperature- 150°C, sampling cone- 45, extraction cone- 4.5, source gas flow- 30 mL/min, desolvation temperature- 350°C, cone gas flow- 30 L/Hr and desolvation gas flow- 800 L/Hr. The TOF MS setup includes a Da range from 50 to 2,000 Da and a scan time of 0.5 s. The raw data were processed by Waters MassLynx 4.1 peptide editor software to get the complete integrated sequence of the sample. The individual peptide MS/MS spectra were matched to the database sequence with the help of the ProteinLynx Global Server (PLGS) software (Waters). The peptides were loaded with buffer A and eluted with buffer B (95% acetonitrile, 0.1% formic acid) at a flow rate of 0.3 ml/min.

### MS data processing

Raw data were generated and processed by MassLynx 4.1 (Waters). The individual peptide MS/MS spectra were matched to the UniProt *Bos taurus* reference proteome database sequence for protein identification on the PLGS software (Waters). The parameters used for the identification are as follows: peptide mass tolerance at the MS1 level, 50 ppm; fragment mass tolerance at the MS2 level, 100 ppm; minimum number of fragment matches for peptides, 2; minimum number of fragment matches for proteins, 5; minimum number of peptide matches for proteins, 1; and missed cleavages, 1. Carbamidomethyl on cysteine as fixed modification and oxidation of methionine and N-terminal acetylation were considered as variable modifications for database search. The score is calculated by the expression analysis extension of the PLGS software based on the relevance of the protein present in both samples being compared. Differentially expressed proteins were identified by calculating the fold change of expression values (log base2) with respect to the control samples. Differentially expressed proteins include the proteins with higher abundance (< −1 fold) and lower abundance (< −1 fold) in the treated sample. The unique proteins mentioned for each control and treated are the proteins that did not have any matching peptides or *m*/*z* values between the groups.

### Gene ontology and pathway analysis of proteins

The functional annotation of the differentially expressed proteins was performed using advanced bioinformatics tools available online. Web sources like Database for Annotation, Visualization and Integrated Discovery (DAVID) version 6.8 were utilized for functional annotation and pathway analysis. Gene names were uploaded in the abovementioned software for gene ontology (GO) analysis. Furthermore, Cytoscape 3.7.1, an open-source software platform with the ClueGO plugin, was used for the visualization of protein–protein interaction networks and biological pathways at the molecular level.

## Results

### Global proteomic profile of fresh, equilibrated, and cryopreserved spermatozoa

**Figure 1:**
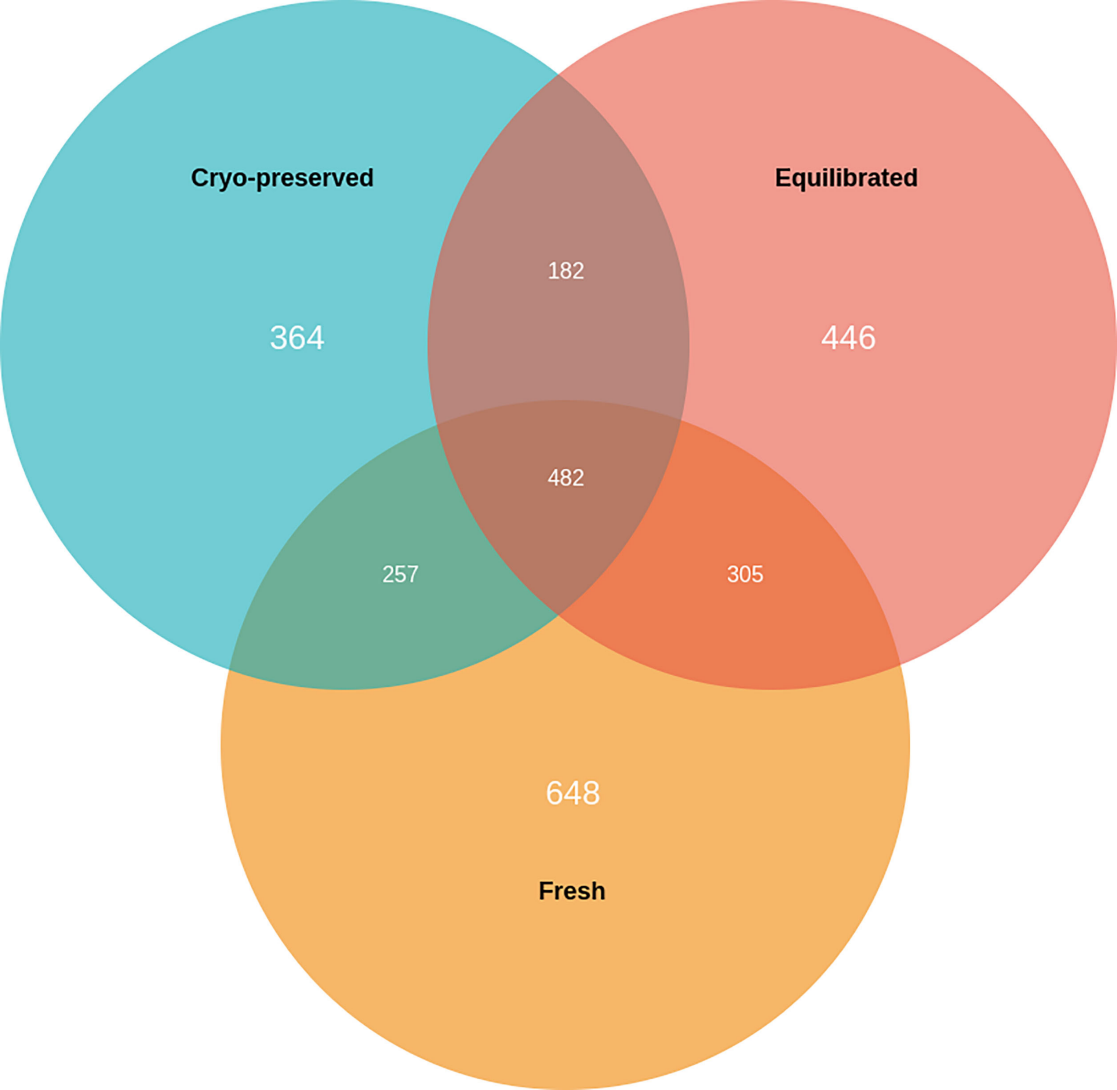
Venn diagram showing the number of unique and common proteins present in fresh, equilibrated, and cryopreserved semen samples.

**Figure 2:**
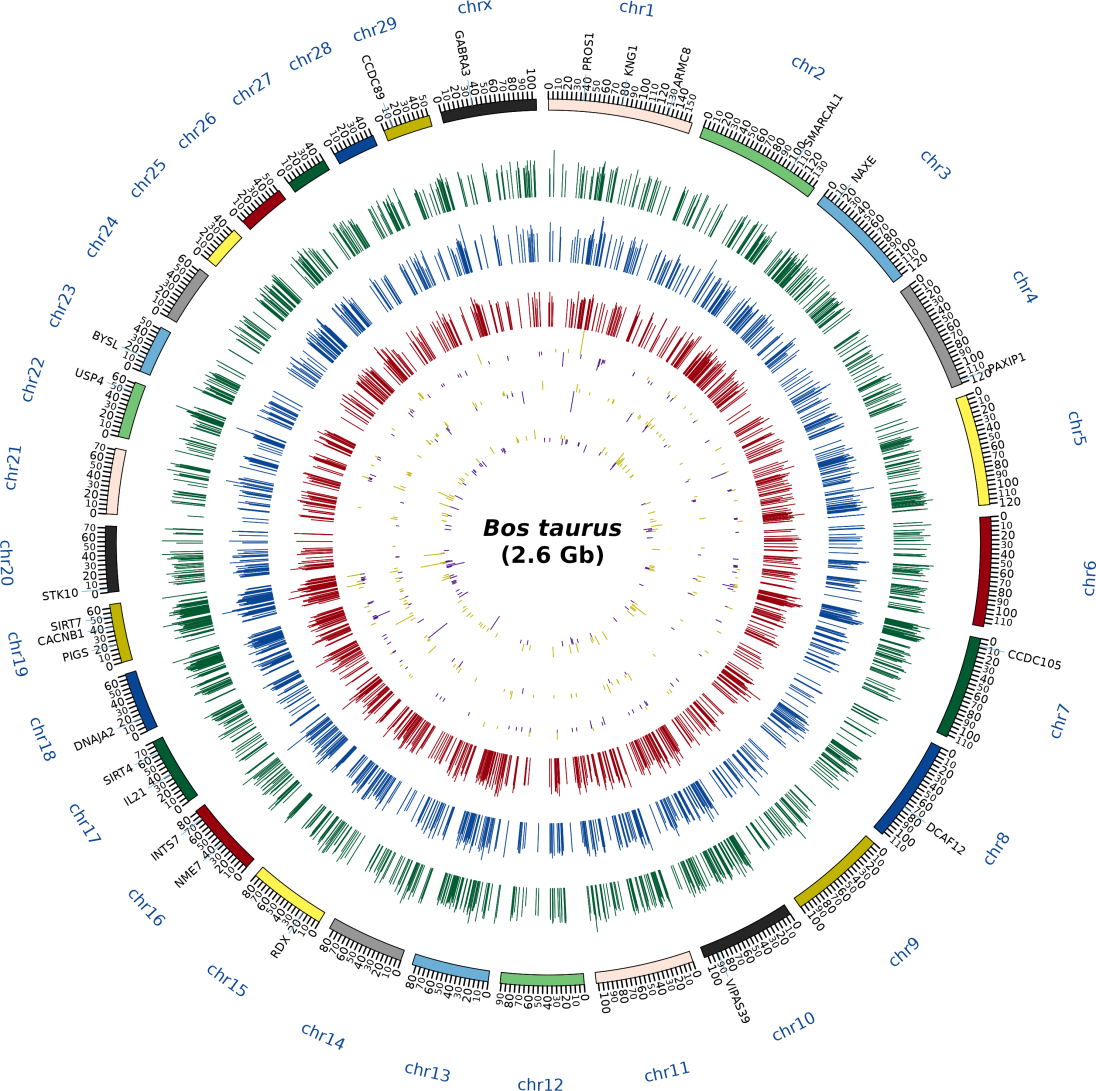
Circos plot depicting the chromosome coverage and protein expression during the different stages of cryopreservation. The outermost circle (first layer) contains the names of the abundantly dysregulated proteins. The next circle (second layer) corresponds to the chromosomal bands, the green histogram (third layer) shows the expression of the proteins in the freshly ejaculated sperm, the blue histogram (fourth layer) indicates the expression of the proteins in the equilibrated sperm, and the red histogram (fifth layer) indicates the expression of the proteins in the cryopreserved sperm. Subsequently, the differentially expressed proteins between fresh and equilibrated sperm (sixth layer), the differentially expressed proteins between equilibrated and cryopreserved sperm (seventh layer), and the differentially expressed proteins between fresh and cryopreserved sperm (eighth layer) were indicated in yellow (detected in higher abundance) and purple (detected in lower abundance) colors.

A total of 1,692, 1,415, and 1,285 proteins were detected in fresh, equilibrated, and cryopreserved spermatozoa, respectively. Among these, 462 proteins were common to all three groups, while 648, 446, and 364 proteins were identified as unique to fresh, equilibrated, and cryopreserved spermatozoa, respectively (**Figure 1**). It was observed that 905 and 751 proteins were found to be lost during equilibration and ultralow freezing, respectively. The proteins were mapped to the chromosome, and their expression in fresh, equilibrated, and cryopreserved sperm and their differential pattern among the groups are shown in **Figure 2**.

### Gene ontology classification and pathway analysis of proteins detected in fresh, equilibrated, and cryopreserved spermatozoa

**Figure 3:**
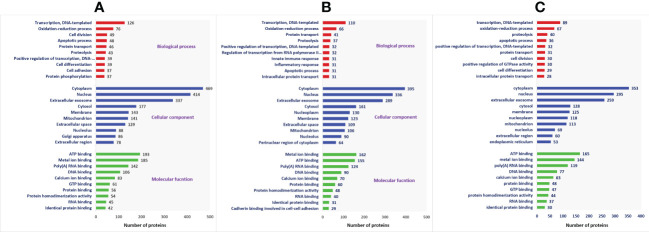
Functional classification of proteins of fresh **(A)**, equilibrated **(B)**, and cryopreserved **(C)** spermatozoa based on gene ontology terms (biological process, cellular component, molecular function).

A total of 1,692 proteins were detected in fresh spermatozoa. DAVID software was used for gene ontology enrichment analysis, which revealed that transcription (3.53%), cytoplasm (13.14%), and ATP binding (5.41%) were the most predominant GO terms in the biological process, cellular component, and molecular function, respectively. The gene ontology of the fresh sperm proteins is depicted in **Figure 3A**. In equilibrated spermatozoa, 1,415 proteins were detected and the gene ontology analysis revealed that transcription (3.60%), cytoplasm (12.93%), and metal ion binding (5.30%) are the most predominant GO terms in equilibrated spermatozoa. The gene ontology of the equilibrated sperm proteins is depicted in **Figure 3B**. In cryopreserved spermatozoa, 1,285 proteins were detected and gene ontology analysis revealed that transcription (3.23%), cytoplasm (12.79%), and ATP binding (5.98%) were the most predominant GO terms. The gene ontology of the cryopreserved sperm proteins is depicted in **Figure 3C**. The protein profiles of equilibrated and cryopreserved spermatozoa indicated a reduction in the number of proteins involved in all biological processes, cellular components, and molecular functions as compared with fresh spermatozoa.

**Figure 4:**
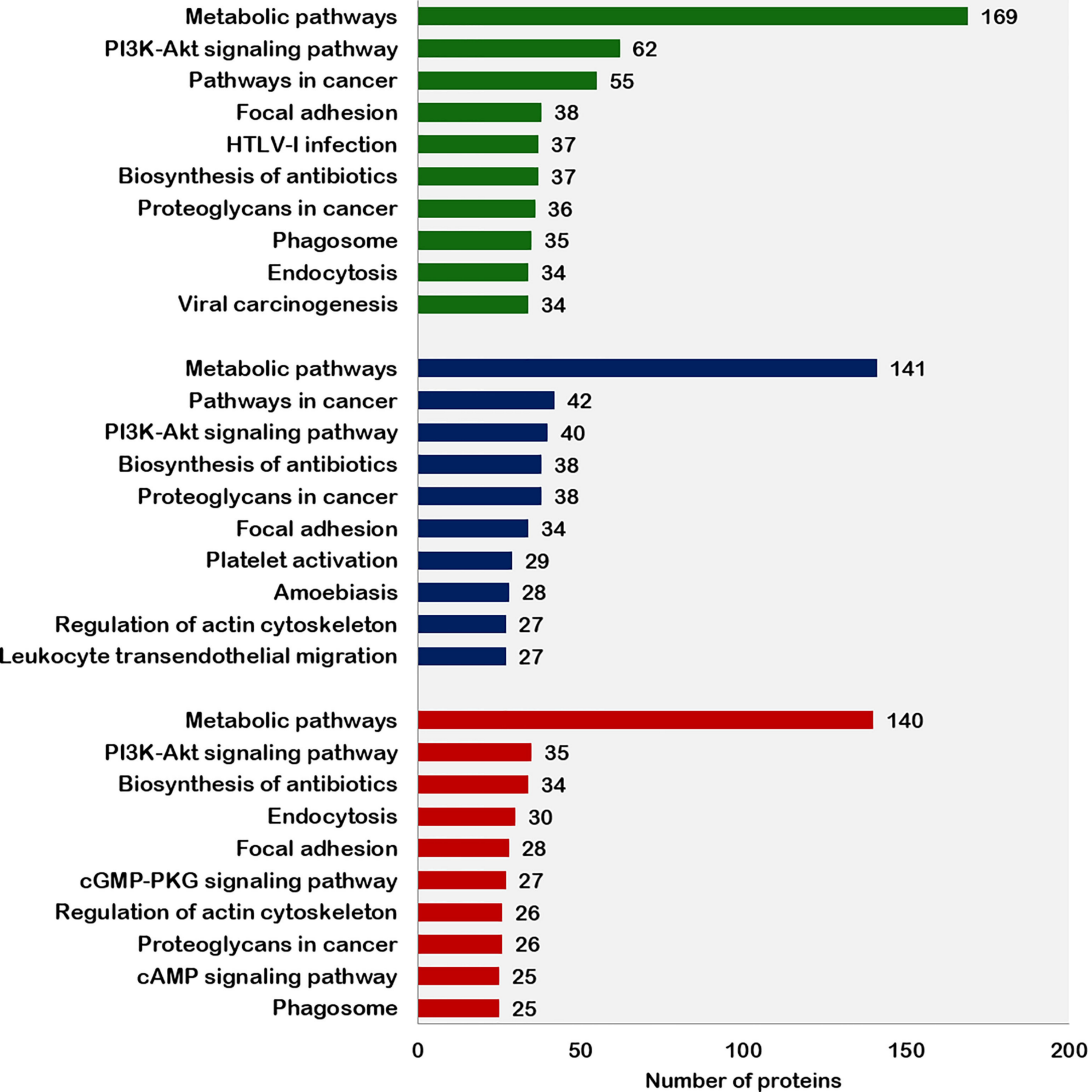
Top 10 pathways enriched with proteins from fresh, equilibrated, and cryopreserved spermatozoa.

The top 10 pathways associated with the proteins expressed in fresh, equilibrated, and cryopreserved spermatozoa are depicted in **Figure 4**. Pathway enrichment of fresh sperm proteins (1,692) revealed the involvement of 1,079 proteins in 103 different pathways. Among these pathways, metabolic pathways and the PI3K–Akt signaling pathways were the two crucial pathways identified in fresh spermatozoa. Pathway enrichment of sperm proteins (1,415) in equilibrated spermatozoa revealed that 444 proteins were involved in 86 different pathways. The number of proteins involved in all pathways, including the metabolic pathways and the PI3K–Akt signaling pathway, was significantly lower than in fresh spermatozoa. Pathway enrichment of cryopreserved sperm proteins (1,285) revealed that 438 proteins were involved in 87 different pathways. The number of proteins involved in all enrichment pathways including major pathways was decreased as compared with fresh and equilibrated spermatozoa.

### Differentially expressed proteins between fresh and equilibrated spermatozoa

**Figure 5:**
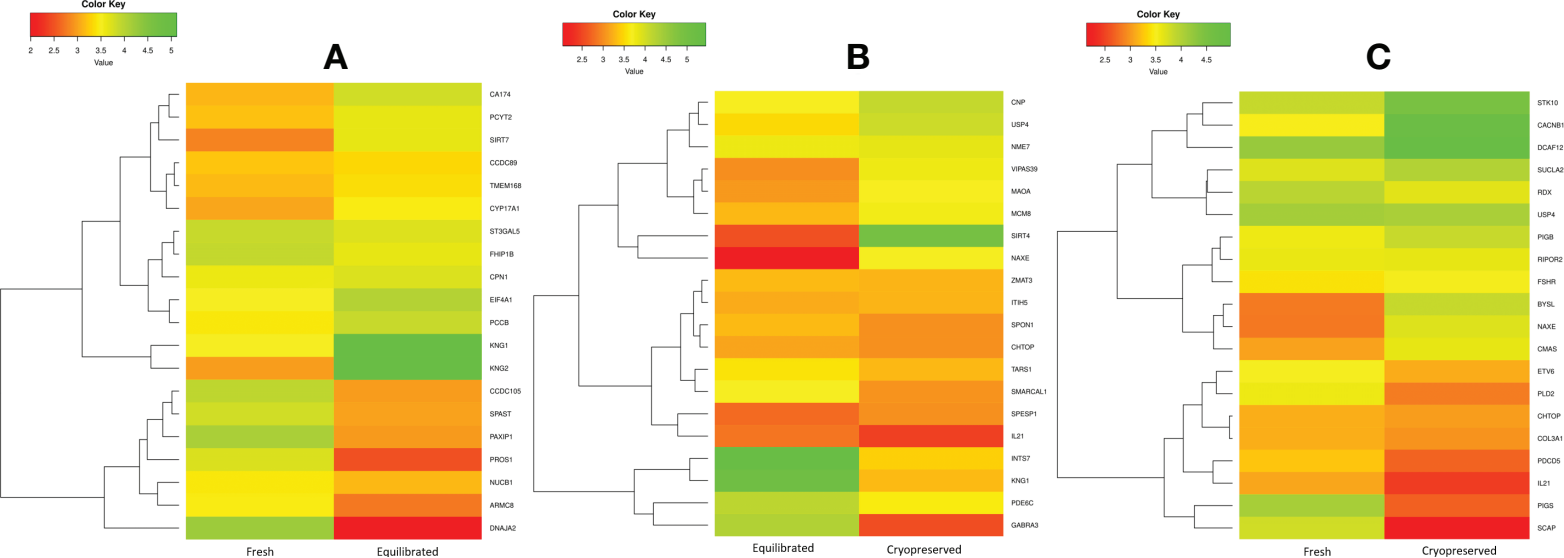
The heatmap of the top 10 abundantly differentially expressed proteins based on their expression intensities. **(A)** Differentially expressed proteins between fresh and equilibrated sperm, **(B)** differentially expressed proteins between equilibrated and cryopreserved sperm, and **(C)** differentially expressed proteins between fresh and cryopreserved sperm.

**Figure 6:**
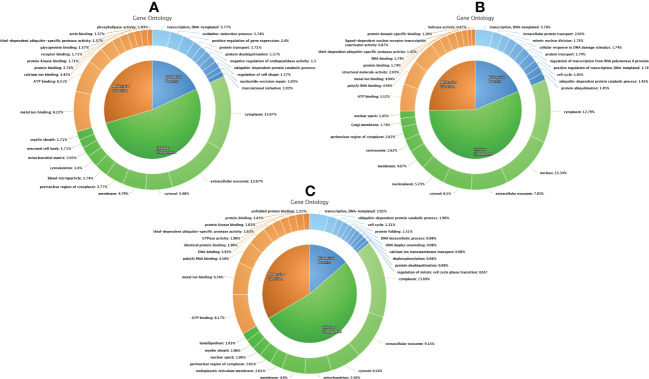
Functional classification of differentially expressed proteins between fresh and equilibrated spermatozoa **(A)**, differentially expressed proteins between equilibrated and cryopreserved sperm **(B)**, and differentially expressed proteins between fresh and cryopreserved sperm **(C)**.

A total of 166 proteins were differentially expressed in equilibrated spermatozoa as compared with fresh spermatozoa. Of these, 61 proteins were in higher abundance and 105 proteins were in lower abundance. The heatmap of the top 10 abundantly dysregulated proteins based on their expression intensities is shown in **Figure 5A**. The top 10 proteins with higher abundance and lower abundance in equilibrated sperm compared with fresh sperm and their fold change are shown in **Supplementary Table 1**. The gene ontology of the differentially expressed proteins between fresh and equilibrated spermatozoa is depicted in **Figure 6A**. Pathway analysis of differentially expressed proteins revealed the involvement of 166 differentially expressed proteins (DEPs) in 26 different pathways. The top 10 dysregulated pathways and associated proteins are depicted in **Table 1**. Among the 26 dysregulated pathways, the actin cytoskeleton pathway, which is related to spermatogenesis, is highly dysregulated in equilibrated spermatozoa as compared with fresh spermatozoa.

**Table 1:** Top 10 dysregulated pathways between fresh and equilibrated spermatozoa and the associated proteins.

Pathways	Protein count	Proteins involved
Metabolic pathways	18	ODC1, PNLIPRP2, MAOA, CS, TKT, LTC4S, DSE, GLCE, ALAS1, CYP17A1, PLCB4, ST3GAL5, IDH1, GPT, PCYT2, PCCB, ALDH9A1, DEGS1
Pathways in cancer	8	PLCB4, ADCY7, STAT5B, ITGA3, KIT, STAT3, PIK3R2, TGFB2
Complement and coagulation cascades	6	KNG1, A2M, FGB, C1S, F7, PROS1
Amoebiasis	6	PLCB4, TLR2, ITGB2, ACTN2, PIK3R2, TGFB2
Chemokine signaling pathway	6	PLCB4, ADCY7, NCF1, STAT5B, STAT3, PIK3R2
Proteoglycans in cancer	6	TLR2, RDX, MSN, STAT3, PIK3R2, TGFB2
Biosynthesis of antibiotics	6	ODC1, CS, IDH1, TKT, PCCB, ALDH9A1
Regulation of actin cytoskeleton	6	ITGB2, RDX, ACTN2, ITGA3, MSN, PIK3R2
Pancreatic secretion	5	PNLIPRP2, CLCA1, PLCB4, ADCY7, SLC4A4
Carbon metabolism	5	CS, IDH1, GPT, TKT, PCCB

### Differentially expressed proteins between equilibrated and cryopreserved spermatozoa

A total of 147 proteins were differentially expressed in cryopreserved spermatozoa as compared with equilibrated spermatozoa. Of these, 104 proteins were in higher abundance and 43 proteins were in lower abundance in cryopreserved spermatozoa compared with equilibrated sperm. The heatmap of the top 10 abundantly dysregulated proteins based on their expression intensities is shown in **Figure 5B**. The top 10 proteins with higher abundance and lower abundance in cryopreserved sperm compared with equilibrated sperm and their fold change are shown in **Supplementary Table 2**. The gene ontology of the differentially expressed proteins between equilibrated and cryopreserved spermatozoa is depicted in **Figure 6B**. Pathway analysis of DEPs revealed the involvement of 147 DEPs in 6 different pathways, which are depicted with associated proteins in **Table 2**.

**Table 2:** Dysregulated pathways between equilibrated and cryopreserved spermatozoa and the associated proteins.

Pathways	Protein count	Proteins involved
Biosynthesis of antibiotics	5	ODC1, GFPT2, HK1, ACLY, NME7
Viral carcinogenesis	5	YWHAG, YWHAH, DDB1, YWHAB, ACTN2
Amino sugar and nucleotide sugar metabolism	4	GNPDA1, CMAS, GFPT2, HK1
Cell cycle	4	YWHAG, YWHAH, YWHAB, MCM3
Hippo signaling pathway	4	YWHAG, YWHAH, YWHAB, CDH1
Aminoacyl-tRNA biosynthesis	3	YARS, SARS, CARS2

### Differentially expressed proteins between fresh and cryopreserved spermatozoa

A total of 156 proteins were differentially expressed between fresh and cryopreserved spermatozoa. Of these, 84 proteins were in higher abundance and 72 proteins were in lower abundance in equilibrated spermatozoa. The heatmap of the top 10 abundantly dysregulated proteins based on their expression intensities is shown in **Figure 5C**. The top 10 proteins with higher abundance and lower abundance in cryopreserved sperm compared with fresh sperm and their fold change are shown in **Supplementary Table 3**. The gene ontology of the DEPs between fresh and cryopreserved spermatozoa is depicted in **Figure 6C**. Pathway analysis of DEPs revealed the involvement of 156 DEPs in 12 different pathways, in which the top 10 pathways are depicted in **Table 3**. Among the dysregulated pathways, the metabolic pathway and the nucleotide excision repair pathway were found to be more specific. The proteins involved in the nucleotide excision repair pathway, which is related to DNA damage, were detected in higher abundance.

**Table 3:** Top 10 dysregulated pathways between fresh and cryopreserved spermatozoa and the associated proteins.

Pathways	Protein count	Proteins involved
Metabolic pathways	25	PLD2, PNLIPRP2, FUT8, CMAS, MAOA, MAOB, ACLY, PIGS, LTC4S, PSPH, ACSBG1, DGKA, ATP6V1A, DHRS3, ST3GAL5, POLE3, ALDH1B1, GADL1, NT5C2, PIGB, ADSL, ATP6V0A1, SUCLA2, UGP2, DEGS1
Biosynthesis of antibiotics	6	ALDH1B1, ADSL, ACLY, PSPH, SUCLA2, UGP2
Purine metabolism	5	GUCY2F, POLE3, PDE5A, NT5C2, ADSL
Prostate cancer	4	HSP90B1, HSP90AA1, PIK3CA, CTNNB1
Estrogen signaling pathway	4	HSP90B1, HSP90AA1, GNAO1, PIK3CA
Histidine metabolism	3	ALDH1B1, MAOA, MAOB
DNA replication	3	DNA2, POLE3, MCM3
Glycine, serine, and threonine metabolism	3	MAOA, MAOB, PSPH
Nucleotide excision repair	3	POLE3, DDB1, ERCC2
Tryptophan metabolism	3	ALDH1B1, MAOA, MAOB

**Figure 7:**
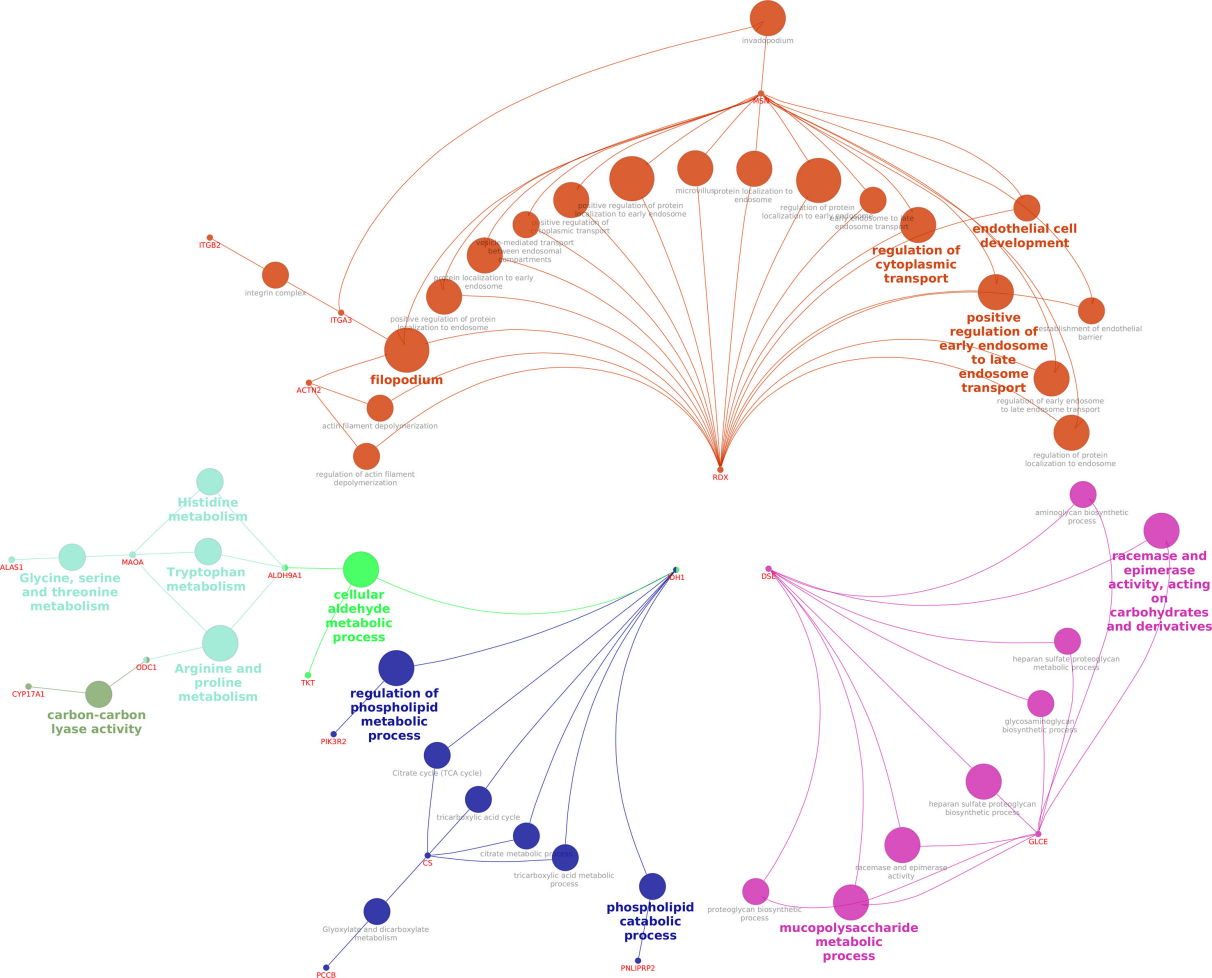
Interaction network of DEPs between fresh and equilibrated spermatozoa.

The network interaction of DEPs between fresh and equilibrated spermatozoa is shown in **Figure 7**. It was observed that DEPs were interacting with each other and involved mainly in metabolism-related processes such as the regulation of the phospholipid metabolic process and the mucopolysaccharide metabolic process.

## Discussion

The influence of the cryopreservation process on the bull sperm proteome is less explored. Studies conducted on human (26, 27), boar (24, 28), ram (29), rabbit (30), and rooster (31) showed that the process of cryopreservation may induce quantitative changes in sperm proteome. These studies indicate that the cryopreservation process alters the abundance levels of sperm proteins related to membrane permeability, metabolism, flagella structure, motility, intracellular signaling, capacitation, apoptosis, and fertilization. However, the possible cause of sperm proteome alteration and the stage of cryopreservation which is highly responsible for sperm proteomic changes are not fully understood. We report here that most of the quantitative proteome and functional pathway alterations occurred during the equilibration stage as compared with the ultralow freezing process.

In our study, the 905 and 751 proteins observed in freshly ejaculated sperm were not detected after equilibration and ultralow freezing, respectively, indicating that the process of equilibration may cause more alterations in the sperm protein profile than the ultralow freezing process. Although the precise reason for such loss of proteins during equilibration is not explainable with the available knowledge, it is possible that the proteins may be lost from spermatozoa during the equilibration process due to shedding, active cleavage, or degradation. However, such mechanisms have not been investigated fully. It has been reported that cold denaturation of protein occurs at a temperature range of 0°C to −20°C, and globular proteins often undergo partially reversible denaturation. Denaturation of proteins during cryopreservation results in the leakage of protease from the lysosomes found in the acrosome leading to loss of membrane integrity (32). During cryopreservation, some proteases and protease inhibitors are prematurely activated; thus, natural proteolysis regulation in cryopreserved semen is compromised (33). This may also be due to the heterogenicity of seminal plasma and sperm in the mammalian ejaculate (34) The protein changes in sperm may also be due to the difference in their responsiveness to seminal plasma (35). Another possible reason, especially important to intracellular proteins, is the increased membrane sensitivity that renders these proteins to be made more accessible to lysates inside the frozen spermatozoa, which could possibly be involved in protein degradation (36). These studies support our findings on sperm protein loss during cryopreservation with possible reasons such as the shedding of proteins through an altered membrane and proteolysis. However, this may be due to the presence of these proteins in low abundance below the detection level. Despite all these factors, extenders also could affect the expression of proteins. Egg yolk-based extenders are commonly used for bull sperm cryopreservation; however, due to sanitary concerns, there has been a tendency against using egg yolk in cryoprotective media in recent years. The various extenders had affected the sperm proteins (37), and it has also been shown that altered membrane permeability enables the shedding of proteins from the spermatozoa (38). Similar to previous bull sperm protein studies (39), we also used an egg yolk-free commercial extender in this study.

On the other hand, we observed that 446 and 364 proteins that were not detected in freshly ejaculated sperm were detected after equilibration and ultralow freezing, respectively. Similar to our findings, Wang et al. found 22 proteins that became more abundant during cryopreservation, and the vast majority of them were functionally classified as intracellular proteins. The possible causes of equilibration-induced proteomic profile alteration would be cryoprotectant-induced osmotic stress which causes cell shrinkage and irreversible alteration in sperm membrane integrity (40) and potential binding of surrounding seminal plasma proteins to the spermatozoa (41). As seminal plasma is an unregulated body fluid unlike blood, its composition hugely varies. The freezability of bull sperm differs based on the difference in the composition of seminal plasma proteins between bulls. Meanwhile, the possible causes of increased protein abundance during ultralow freezing are currently not fully understood (26), although it has been suggested that increased phosphorylation could be the cause of protein abundance following cryopreservation. These proteins might have been expressed in low abundance below the detection level in fresh sperm, but the expression might have increased during cryopreservation. It may be inferred that quantitative proteomic alterations such as both the increase and decrease in protein abundance are more prevalent during equilibration than during the ultralow freezing process.

In order to elucidate the functional differences of proteome among groups, gene ontology analysis of global protein profiles and differential expression of proteins was carried out. Gene ontology analysis of individual global protein profile found that the proteins involved in inevitable biological processes such as sperm metabolism, signal transduction, energy synthesis, proteolysis, protein degradation, and apoptosis were decreased throughout the cryopreservation process. Among the pathways identified, the metabolic pathway, the PI3K–Akt signaling pathway, and the focal adhesion pathway are the crucial pathways in all three groups. The important metabolic pathways that produce cellular energy in the form of ATP are glycolysis and mitochondrial oxidative phosphorylation (42, 43). ATP is the indispensable fuel for the sperm and the facilitator of sperm motility (44, 45). The hydrolysis of ATP is essential for the sperm flagellar movement to propagate sperm (46). The PI3K–Akt pathway is an intracellular signal transduction pathway in response to extracellular signals, which promotes metabolism, proliferation, cell survival, growth, and angiogenesis (47). This pathway was reported to be critical for sperm motility and mitochondrial ROS generation (48). The focal adhesion pathway is known to form the focal adhesion protein complex in order to activate many intracellular signaling pathways in relation to capacitation and acrosome reaction. Several focal adhesion proteins such as β1-integrin, focal adhesion kinase (FAK), paxillin, vinculin, talin, and α-actinin form a protein complex that plays a vital role in the regulation of acrosomal integrity, polymerization, and remodeling of the actin cytoskeleton (49). Meanwhile, the pathway analysis of equilibrated and cryopreserved sperm proteins flaunted that the number of proteins entangled in the abovementioned crucial pathways was decreased in both equilibrated and cryopreserved spermatozoa, which may result in reduced signal transduction, energy synthesis, and acrosomal integrity in cryopreserved spermatozoa.

We found 166 DEPs during the equilibration process (vs. fresh sperm), 147 DEPs during the ultralow freezing process (vs. equilibrated sperm), and 156 DEPs throughout the cryopreservation process (vs. fresh sperm), which included both equilibration and ultralow freezing. Among these, the abundance was higher during the equilibration process with 105 proteins as compared with 43 proteins during the ultralow freezing process. The dysregulated proteins were mostly involved in cellular processes, metabolic processes, binding activities, and catalytic activities in relation to sperm function in all three comparisons. Among the biological processes observed, four proteins (USP2, USP4, USP21, and BAP1) involved in the ubiquitin–proteasome system (UPS) such as deubiquitination and ubiquitin-dependent protein catabolic process were highly abundant after the equilibration process. Ubiquitination is a post-translational modification involved in transcriptional regulation, embryonic development, preimplantation, cell cycle control, immune response, oncogenesis, apoptosis, intracellular signaling pathways, and DNA repair mechanisms such as histone ubiquitination and ubiquitin-dependent protein catabolic process in DNA damage (50). Deubiquitination is involved in protein degradation and is the terminal stage of the apoptotic process (51). Excessive levels of ubiquitinated proteins in spermatozoa represent the dysfunction of the UPS which is negatively correlated with sperm motility, morphology, and chromatin integrity (52, 53). The involvement of the UPS in fertilization is supported by substantial scientific evidence. These proteasomes are necessary for the sperm to complete the zona penetration (54–59). The CYB5R4 (Cytochrome B5 reductase) protein is involved in stress-induced ROS production (60), and the LOC784768 (Calcium-activated chloride channel) protein plays a crucial role in acrosome reaction, capacitation, and sperm motility (61). The increased abundance of these proteins during equilibration is associated with premature acrosome reaction and capacitation, which might be initiated during the equilibration process itself. Among the molecular functions observed, calcium ion binding is an essential criterion for the sperm to complete its normal capacitation and acrosome reaction (62). Among the 24 proteins commonly dysregulated in both equilibrated and cryopreserved sperm as compared with fresh sperm, the CDC37 (63), ERCC2 (64), AP2M1 (65), USP2 (58), and MAOA (66) were related to male fertility. Interestingly, all these five proteins were in lower abundance after equilibration and persisted in lower levels even after cryopreservation as compared with fresh sperm.

The pathway enrichment analysis of dysregulated/differentially expressed proteins revealed that 26, 6, and 12 pathways were altered during equilibration, ultralow freezing, and throughout the cryopreservation process, respectively. Among these pathways, the metabolic pathway and the regulation of the actin cytoskeleton pathway were found to be related to sperm functions. Though the metabolic pathway was dysregulated in both equilibrated and cryopreserved sperm, the expression of the majority of proteins (ODC1, PNLIPRP2, MAOA, TKT, DSE, GLCE, PLCB4, ST3GAL5, IDH1, and PCCB) involved in this pathway was detected in lower abundance during the equilibration process. Fu et al. (67) also reported that metabolism-related proteins involved in glyoxylate and dicarboxylate metabolism, glycolysis/gluconeogenesis, and pyruvate metabolism were altered during cryopreservation. Metabolic pathways play a major role in energy production to maintain cellular processes like motility, hyperactivation, capacitation, and acrosome reaction (68). Spermatozoa require glycolytic energy for motility, capacitation, and acrosome reaction and mitochondrial OXPHOS for sperm cell differentiation and maturation (42, 69). An important finding of our study is that the proteins involved in major metabolic pathways such as the tricarboxylic acid (TCA) cycle, glycolysis, and the inositol phosphate pathway were in lower abundance in equilibrated spermatozoa. Since metabolic pathways play an important role in sperm functional attributes, the lower abundance levels of proteins involved in metabolic pathways may have a big impact on the ability of the sperm to fertilize. Lower abundance or loss of proteins involved in the metabolic pathway could be associated with ATP deficiency, which may lead to reduced sperm motility. Citrate synthase, an enzyme protein of the TCA cycle, delays sperm–egg fusion by initiating Ca^2+^ oscillation and negatively correlates with fertility (70), but its higher abundance during equilibration may reduce the fertility of the spermatozoa. Regarding the actin cytoskeleton pathway, the presence of actin polymerization in the tail region is necessary for sperm motility during post-testicular maturation (71) and sperm oocyte incorporation (72), and actin polymerization in the acrosomal region is necessary for capacitation and acrosomal reaction (73). The cytoskeletal proteins F-actin and β-dystrobrevin were altered by the cryopreservation process, allowing them to become more delicate (74). Similarly, Yoon et al. (75) reported that cryopreservation alters the ephrinR-actin, actin cytoskeleton assembly, and actin cytoskeleton regulatory mechanisms in epididymal spermatozoa. In our study, we observed that out of the six proteins involved in the actin cytoskeleton pathway, five proteins were detected in lower abundance in equilibrated spermatozoa, and there was no evidence of alteration during the ultralow freezing process. Therefore, we showed here that proteins involved in vital pathways such as the metabolic pathways and the regulation of the actin cytoskeleton pathway were dysregulated during the equilibration stage of cryopreservation itself.

Collectively, the quantitative and qualitative alterations in sperm proteins, which are involved in functional attributes and pathways associated with energy metabolism, motility, capacitation, and sperm membrane stability, were observed during the equilibration stage of cryopreservation. Earlier, it was thought that the sperm cryodamage during cryopreservation was due to exposure of the sperm to ultralow temperature (−196°C); however, the current study reports that the majority of the damages to sperm proteins are happening during the equilibration stage itself. Therefore, it is critical to understand how the equilibration process differs from ultralow freezing in terms of how it impacts essential sperm functional attributes, in order to tailor the cryopreservation protocols with the primary aim of preserving sperm proteome architecture by incorporating specific additives. Accordingly, the protein alterations can be minimized in the spermatozoa during cryopreservation to achieve high conception rates with frozen-thawed spermatozoa.

## Data availability statement

The datasets presented in this study can be found in online repositories. The names of the repository/repositories and accession number(s) can be found below: https://repository.jpostdb.org/entry/JPST001500, PXD031895.

## Ethics statement

The animal study was reviewed and approved by Institute Animal Ethics Committee.

## Author contributions

RA: methodology, experiment, writing—original draft, and data curation. AK: conceptualization, project administration, supervision, funding, and writing—review and editing. MS and JK: data curation and bioinformatics analysis. PN and TK: methodology and data curation. KE: methodology and writing—original draft. RB: samples and methodology. TM: samples and methodology. RK: formal analysis and writing—review and editing. TD: formal analysis and writing—review and editing. All authors contributed to the article and approved the submitted version.
