# Supplementary material for: The cryopreservation process induces alterations in proteins associated with bull sperm quality: The equilibration process could be a probable critical control point
Source: Front Endocrinol (Lausanne). 2022 Dec 9;13:1064956. doi: 10.3389/fendo.2022.1064956 (PMC9787546; doi:10.3389/fendo.2022.1064956)
Supplement: Supplementary file 1 [file DataSheet_1.docx]

**Supplementary figure 1: Quality check of extracted proteins using SDS-PAGE**

(Sample ID: A – Fresh spermatozoa; B – Equilibrated spermatozoa and C – Cryopreserved spermatozoa)
