# Supplementary material for: The cryopreservation process induces alterations in proteins associated with bull sperm quality: The equilibration process could be a probable critical control point
Source: Front Endocrinol (Lausanne). 2022 Dec 9;13:1064956. doi: 10.3389/fendo.2022.1064956 (PMC9787546; doi:10.3389/fendo.2022.1064956)
Supplement: Supplementary file 2 [file DataSheet_2.docx]

**Supplementary Table 1. Top 10 proteins with higher abundance and top 10 proteins with lower abundance in equilibrated spermatozoa compared to fresh spermatozoa**

| **Proteins with higher abundance (>1)** | | | |
| --- | --- | --- | --- |
| **S.No** | **Symbol** | **Protein name** | **Fold change** |
| 1. | KNG2 | Kininogen-2 | 10.95 |
| 2. | KNG1 | Kininogen-1 | 10.41 |
| 3. | IF4A1 | Eukaryotic initiation factor 4A-I | 6.34 |
| 4. | SIR7 | NAD-dependent protein deacetylase sirtuin-7 | 5.42 |
| 5. | CCD89 | Coiled-coil domain-containing protein 89 | 4.15 |
| 6. | PCYT2 | Ethanolamine-phosphate cytidylyltransferase | 3.64 |
| 7. | C1orf174 | UPF0688 protein C1orf174 homolog | 3.42 |
| 8. | CPN1 | Carboxypeptidase N catalytic chain | 3.00 |
| 9. | CYP17A1 | Steroid 17-alpha-hydroxylase/17,20 lyase | 2.81 |
| 10. | TMEM168 | Transmembrane protein 168 | 2.74 |
| **Proteins with lower abundance (<-1)** | | | |
| 1. | DNAJA2 | DnaJ homolog subfamily A member 2 | -7.21 |
| 2. | PAXIP1 | PAX-interacting protein 1 | -6.14 |
| 3. | PROS1 | Vitamin K-dependent protein S | -5.65 |
| 4. | ARMC8 | Armadillo repeat-containing protein 8 | -4.77 |
| 5. | CCDC105 | Coiled-coil domain-containing protein 105 | -4.51 |
| 6. | SPAST | Spastin | -3.26 |
| 7. | PCCB | Propionyl-CoA carboxylase beta chain, mitochondrial | -3.17 |
| 8. | FAM160A2 | FTS and Hook-interacting protein | -3.12 |
| 9. | ST3GAL5 | Lactosylceramide alpha-2,3-sialyltransferase | -3.09 |
| 10. | NUCB1 | Nucleobindin-1 | -2.60 |

**Supplementary Table 2.** **Top 10 proteins with higher abundance and top 10 proteins with lower abundance in cryopreserved spermatozoa compared to equilibrated spermatozoa**

| **Proteins with higher abundance (>1)** | | | |
| --- | --- | --- | --- |
| **S.No** | **Symbol** | **Protein name** | **Fold change** |
| 1. | SIR4 | NAD-dependent protein lipoamidase sirtuin-4, mitochondrial | 8.60 |
| 2. | UBP4 | Ubiquitin carboxyl-terminal hydrolase 4 | 4.95 |
| 3. | NDK7 | Nucleoside diphosphate kinase 7 | 4.13 |
| 4. | SPE39 | Spermatogenesis-defective protein 39 homolog | 4.00 |
| 5. | NNRE | NAD(P)H-hydrate epimerase | 3.98 |
| 6. | MAOA | Amine oxidase [flavin-containing] A | 3.51 |
| 7. | SPESP1 | Sperm equatorial segment protein 1 | 3.46 |
| 8. | MCM8 | DNA helicase MCM8 | 3.39 |
| 9. | ZMAT3 | Zinc finger matrin-type protein 3 | 3.36 |
| 10. | CNP | 2',3'-cyclic-nucleotide 3'-phosphodiesterase | 3.36 |
| **Proteins with lower abundance (<-1)** | | | |
| 1. | INTS7 | Integrator complex subunit 7 | -10.21 |
| 2. | KNG1 | Kininogen-1 | -8.99 |
| 3. | GBRA3 | Gamma-aminobutyric acid receptor subunit alpha-3 | -5.21 |
| 4. | SMAL1 | SWI/SNF actin-dependent regulator of chromatin subfamily A-like protein 1 | -3.97 |
| 5. | CHTOP | Chromatin target of PRMT1 protein | -3.43 |
| 6. | PDE6C | Cone cGMP-specific 3',5'-cyclic phosphodiesterase subunit alpha' | -3.36 |
| 7. | SPON1 | Spondin-1 | -2.89 |
| 8. | IL21 | Interleukin-21 | -2.81 |
| 9. | TARS | Threonine--tRNA ligase, cytoplasmic | -2.61 |
| 10. | ITIH5 | Inter-alpha-trypsin inhibitor heavy chain | -2.25 |

**Supplementary Table 3. Top 10 proteins with higher abundance and top 10 proteins with lower abundance in cryopreserved spermatozoa compared to fresh spermatozoa**

| **Proteins with higher abundance (>1)** | | | |
| --- | --- | --- | --- |
| **S.No** | **Symbol** | **Protein name** | **Fold change** |
| 1. | CACB1 | Voltage-dependent L-type calcium channel subunit beta-1 | 8.43 |
| 2. | STK10 | Serine/threonine-protein kinase 10 | 6.87 |
| 3. | DCAF12 | DDB1- and CUL4-associated factor 12 | 5.61 |
| 4. | BYSL | Bystin | 5.53 |
| 5. | NAXE | NAD(P)H-hydrate epimerase | 4.11 |
| 6. | USP4 | Ubiquitin carboxyl-terminal hydrolase 4 | 3.65 |
| 7. | CMAS | N-acylneuraminate cytidylyltransferase | 3.51 |
| 8. | SUCLA2 | Succinate--CoA ligase [ADP-forming] subunit beta, mitochondrial | 3.32 |
| 9. | PIGB | GPI mannosyltransferase 3 | 3.17 |
| 10. | FSHR | Follicle-stimulating hormone receptor | 3.07 |
| **Proteins with lower abundance (<-1)** | | | |
| 1. | PIGS | GPI transamidase component PIG-S | -5.96 |
| 2. | RADI | Radixin | -5.24 |
| 3. | IL21 | Interleukin-21 | -3.20 |
| 4. | CHTOP | Chromatin target of PRMT1 protein | -2.91 |
| 5. | PLD2 | Phospholipase D2 | -2.90 |
| 6. | SCAP | Sterol regulatory element-binding protein cleavage-activating protein | -2.83 |
| 7. | RIPOR2 | Rho family-interacting cell polarization regulator 2 | -2.70 |
| 8. | ETV6 | Transcription factor ETV6 | -2.67 |
| 9. | COL3A1 | Collagen alpha-1(III) chain | -2.45 |
| 10. | PDCD5 | Programmed cell death protein 5 | -2.29 |
